# Telemedicine in community shelters: possibilities to improve chronic care among people experiencing homelessness in Hungary

**DOI:** 10.1186/s12939-022-01803-4

**Published:** 2022-12-17

**Authors:** Sándor Békási, Edmond Girasek, Zsuzsa Győrffy

**Affiliations:** 1 Health Center, Hungarian Charity Service of the Order of Malta, Budapest, Hungary; 2 Telemedicine Workgroup, FitPuli Kft, Győr, Hungary; 3 Institute of Behavioural Sciences, Faculty of Medicine, Semmelweis University, Budapest, Hungary

**Keywords:** Homelessness, Homeless persons, Community shelter, Digital health, Telemedicine, Telehealth, Health equity

## Abstract

**Background:**

Digital health has expanded during the COVID-19 pandemic, while the exclusion of vulnerable populations with limited access to these technologies widens the gap to receive proper care. There is very little data available on the feasibility of telemedicine solutions regarding the chronic care of homeless persons.

**Methods:**

In our study, 75 participants experiencing homelessness were recruited from four social institutions in Budapest, Hungary. The telecare pilot service consisted of six online consultations with a physician and was available in shelters biweekly. Self-developed questionnaires were used after every online session on the originating and remote sites as well, while a follow-up study was also completed among patients after four to six months of pilot closure. Parameters as frequencies, averages, and percentage distributions were analyzed and two linear regression models were built on explaining the doctors’ and patients’ overall rating of visits.

**Results:**

During the pilot, 92.2% (*n* = 415) of originally planned visits were delivered and 55 clients (73.3%) attended the full program. Both the patients’ and physicians’ overall satisfaction was very high (4.52 and 4.79, respectively, on a 5-point Likert scale) and the patients’ overall rating remained similarly high during the follow-up. Comparing the first and sixth visits, physicians reported significant improvements in almost all aspects. The linear regression models proved that confidence in the patients’ assessment and diagnosis had the most prominent effect on the physicians’ overall rating, while ease of use and lack of communication gaps influenced positively the patients’ rating.

**Conclusion:**

The results suggest that telehealth services represent a promising tool to ensure better care continuity while using shelter infrastructure and on-site assistance might reduce the digital exclusion of people experiencing homelessness.

**Supplementary Information:**

The online version contains supplementary material available at 10.1186/s12939-022-01803-4.

## Background

Telemedical health services are not novel concepts, they were offered in several countries well before the novel coronavirus disease 2019 (COVID-19) pandemic [1, 2]. However, they became widely accepted and used only after access to in-person care was severely restricted due to its association with a significant risk of the severe acute respiratory syndrome coronavirus 2 (SARS-CoV-2) infection [3–7]. At the outbreak of the pandemic, early recommendations and publications regarding the rapid launch of telemedicine services emphasized the role of online consultations and remote patient monitoring in substituting traditional in-person care [8, 9]. The need for an alternative care pathway was also perceived in Hungary but the pandemic hit the country unpreparedly: even the legal framework of telemedicine was not established before 2020 and got enacted during the first wave of the pandemic [10]. These evident needs were also the triggers of our telecare pilot presented in this paper.

As digital health technologies emerged and a paradigm shift toward a hybrid primary care model is envisaged [11], access to and routine use of these online platforms and devices have a strong effect on basic healthcare utilization and are now considered as social determinants of health [12]. According to the Global strategy on digital health 2020–2025 of the World Health Organization (WHO), *“Digital health should be an integral part of health priorities and benefit people in a way that is ethical, safe, secure, reliable, equitable and sustainable”* [13]. However, the ways of harnessing digital health are not obvious in case of vulnerable populations. There is evidence that populations with low socioeconomic status use telemedicine less, most likely due to lower access to the Internet and digital technology, and a lack of digital literacy skills [3, 14, 15]. Supporting that reduction of burdens to technology might increase access, a study reported beneficial effects on telehealth usage through tablet distribution among veterans experiencing homelessness in the USA [16]. Also, in a telemental health program, solving difficulties related to the users’ inexperience in digital technology resulted in a significantly higher completion rate [17].

In Hungary, people experiencing homelessness face severe health challenges. In the last 2 decades, the proportion of older generations among homeless people has substantially increased followed by a higher prevalence of chronic conditions [18]. A Hungarian survey published in 2021 found that the self-reported health status of people experiencing homelessness is significantly worse than the lowest income quintile of the general population. In the same study, both the lower access to primary care visits and lack of regular medication in case of existing chronic diseases underscored the shortcomings of care continuity [19].

The COVID-19 pandemic put the social sector under further serious pressure worldwide. Residents of communal shelters could experience heavy limitations during isolation, curfew, and quarantine restrictions [20, 21], while shelters also represented a high risk of SARS-CoV-2 transmission [22]. A dissociation between social and health services potentially increased health care inaccessibility. The need for integration of such services is also emphasized from a public health perspective [23].

Although there are ongoing efforts to offer telehealth services for people experiencing homelessness, mainly in the USA [24–26], scientific evaluation of these initiatives is extremely rare. A publication of the Boston Health Care for the Homeless Program (Boston, MA, USA) reported successful telehealth case management of homeless persons living with HIV during the COVID-19 pandemic [27]. In a study that evaluated a pre-pandemic telehealth program in an urban drop-in center in South Carolina (USA), the authors found that telehealth services improved access to health care and reduced health inequity. It was also associated with high patient satisfaction and significantly positive feedback from the provider side [28].

In our previous research exploring the attitude of homeless persons towards telecare, we found no difference in their openness compared to a Hungarian reference group [29]. The results also supported that homeless people’s general trust in the healthcare system might contribute to a higher approval of telehealth services.

The main goal of our present project was to determine the feasibility, patient experience, and medical relevance of a telehealth service focusing on the care continuity of chronic conditions among people experiencing homelessness and accommodated in community shelters in Budapest, Hungary. Although there are published telehealth projects that recruited homeless persons and other individuals from underserved populations, the present telehealth pilot was the first-ever study that specifically targeted homeless persons through shelters.

## Methods

### Participants

A total number of 75 adult participants were recruited from four shelters providing mid- and long-term accommodation (with a limit of usually one year that might be extended for one more year) to people experiencing homelessness in Budapest, Hungary. According to the European Typology of Homelessness and Housing Exclusion (ETHOS) classification [30], all four shelters were categorized as 3.2 (temporary accommodation), however, there is a Hungarian tendency that clients use these kinds of shelters for a longer time. Although general healthcare services were available for shelter residents prior to the pilot, their usage was ineffective or underutilized as reported by social workers.

Participation in the telecare pilot was on a voluntary basis. As our clients had never experienced telemedicine and to have a thorough understanding of the service by all potential participants, on-site telecare assistants of the social institutions alongside a written consent form also gave a detailed oral description of the whole project.

For participating in the pilot, only one inclusion criterion was applied: the client had to have at least one pre-existing chronic condition that required regular medical follow-up. Although we did not exclude any disease groups, recruitment was focused on clients with cardiovascular, pulmonary, and metabolic diseases. As tracking medical parameters related to these disease groups was manageable easier with basic medical devices, we assumed that medical decision-making on the remote site could be supported better. Exclusion criteria were the existence of severe cognitive impairment, severe communication disabilities (such as severe hearing or visual impairment), and persistent immobility. These restrictions were applied either for the reason that we were not able to provide mobile devices to cover bedridden clients (the visits were done in a separate room within the shelter facility), or the assistants and the physicians asked it during the preparatory focus group discussion, and we have respected that. They reported that they were not entirely comfortable with treating patients with severe hearing or visual impairments through telemedicine.

### Study design

As the first step, participating shelters were chosen operated either by the Hungarian Charity Service of the Order of Malta or a partner institution. We also recruited on-site assistants from the social teams of these shelters. A previous work experience in the healthcare sector was a requirement for the assistants to have a basic knowledge of care pathways.

The final structure of the study, inclusion and exclusion criteria, documentation and response to possible emergency situations were determined during focus group discussions with the participating physicians (*n* = 3) and on-site assistants (*n* = 4) before the patient recruitment phase.

Recruitment went on for four weeks before the telecare visits started (between Feb 8 and March 7, 2021). Prior to the first telecare visit, a short medical folder of patient history was filled in by each participant and was available for the physicians.

Each participant of the pilot was invited to six online telecare visits biweekly (every two weeks) with a focus on medical management of chronic conditions. The visits took place on an appointment basis and keeping appointments were facilitated by the on-site assistants. Anonymized accounts of popular video call services were used by the care teams. Telemedical health care was provided by three physicians of the Health Center of the Hungarian Charity Service of the Order of Malta (Budapest, Hungary) consisting of two internal medicine specialists and a primary care physician. The visits took place between March 10 and July 30, 2021.

**Fig. 1:**
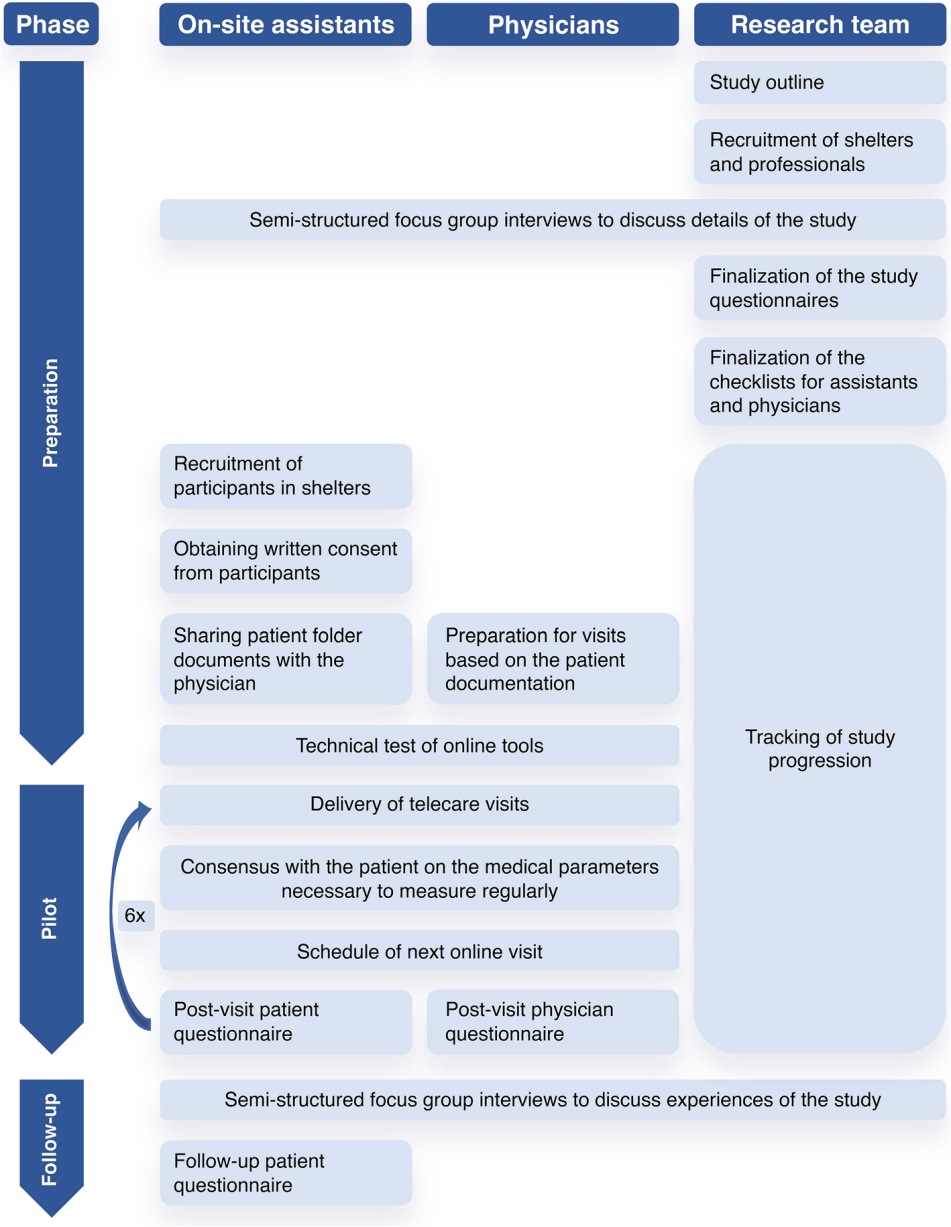
The structure of the telecare study. The figure shows the different activities performed by the stakeholders during the three phases of the study (preparation, pilot, and follow-up) in chronological order

After completion of the pilot, closing focus group discussions were organized for both physicians and on-site assistants to summarize their experiences. A follow-up survey among available previous clients in all four shelters was completed after four to six months of pilot closure, between November 9 and December 7, 2021. The full design of the study is shown in Fig. 1.

### Questionnaires

Both clients on the originating site and physicians on the remote site were asked to complete specific questionnaires regarding their experiences and satisfaction after every online visit. All questionnaires were developed by the research team and fine-tuned after the initial focus group interviews.

The client questionnaire consisted of 11 items and focused on the overall patient experience regarding the telecare visit (on a 5-point Likert scale), the occurrence of any technical difficulties (written description), and feedback on different user aspects of the pilot (e.g., simplicity, comfortability, accountability, and differences to an in-person appointment), also on a 5-point scale. The follow-up client questionnaire consisted of 2 items and asked about the past experience of the pilot and the openness towards participation in a future regular telecare service (both on a 5-point scale).

The physician questionnaire consisted of 14 items focusing on the overall professional experience, technical aspects of the visit, medical relevance, and user experience as a provider. All questionnaires are available both in Hungarian and English versions as additional file [see Additional file 1].

### Ethical considerations and safety

Telemedical healthcare services were provided by the Health Center of the Hungarian Charity Service of the Order of Malta, a provider with legal authorization and extensive experience in the primary care of homeless persons.

Online visits were delivered through anonymized accounts of either Facebook Messenger (Meta Platforms Inc., Menlo Park, CA, USA) or WhatsApp (WhatsApp LLC, Menlo Park, CA, USA) without disclosing personal, medical and any other identifiable data in a written form beyond the video calls. All other documents related to the pilot were handled in a paper and pencil form and were only available to personnel included in the pilot under strict medical secrecy.

Written informed consent was obtained in every case and ethical approval of the pilot study was granted as TUKEB:133/2020 and IV/10,927/2020/EKU by the Scientific Research Ethics Committee of the Medical Research Council of Hungary.

There were no adverse events related to telecare reported during the study.

### Statistical analysis

Data from different sources with anonymous IDs of patients, physicians, and visits were compiled into an analytical database. Data were analyzed with IBM Statistics (SPSS) 27.0 software (IBM, Armonk, NY, USA). As part of the quantitative analysis, we descriptively examined frequencies, averages, and percentage distributions. In comparing averages between groups, we used the ANOVA model and F-test with a *p* < 0.05 significance level, and between variables, we used paired T-test with a *p* < 0.05 significance level. Beyond the descriptive analysis, two linear regression models were built on explaining the doctors’ and patients’ overall rating of telemedicine visits. In these models, the dependent variables were the overall ratings and the explanatory variables were the level of agreement with other statements of physicians and patients, accordingly. In the physicians’ model, the length of the visit, the occurrence of any technical problems, modification of the therapeutic regime, and the measurement of parameters between visits were also involved. In both cases, the linear regression model was executed with the stepwise variable selection method.

## Results

### Demography

75 adult clients participated in the telecare pilot program from four community shelters providing mid- and long-term accommodation. The age distribution of participants was between 44 and 80 years with an average of 62.6 years and a standard deviation (SD) of 7.95. 76.0% (*n* = 57) of the cohort were men, this great majority of male participants corresponded to the distribution of men among people experiencing homelessness in Hungary. Also, lower levels of education (elementary or vocations school) represented more than three-quarters of the sample (*n* = 57, 78.1%). 87.0% (*n* = 60) identified themselves as homeless person, 33.3% (*n* = 20) of them have been homeless for 6–10 years, while almost one-quarter of the cohort (*n* = 14, 23.3%) reported 16 + years of homelessness. Data on nationality was not asked in the demography section as migration background is rarely seen in shelters in Hungary. Regarding healthcare services, 70.8% (*n* = 46) used healthcare services semi-annually or more frequently and participants reported an average of 2.64 (SD = 1.55) chronic conditions. The most common diseases were hypertension (*n* = 60, 80.0%), chronic obstructive pulmonary disease (COPD) (*n* = 28, 37.3%), chronic heart failure (*n* = 23, 30.7%), and diabetes mellitus (*n* = 22, 29.3%). The demographic profile of the sample is shown in Table 1.

**Table 1:** The demographic profile of the sample

Shelter		HCSOM		SHF		BIC 1		BIC 2		Total	
**Number of participants**		12		21		12		30		**75**	
**Age**		***n***	**mean**	***n***	**mean**	***n***	**mean**	***n***	**mean**	***n***	**mean**
		12	56.9	21	63.6	12	64.8	30	63.2	**75**	**62.6**
		***n***	**%**	***n***	**%**	***n***	**%**	***n***	**%**	***n***	**%**
**Gender**	Male	12	100	14	33.3%	7	58.3%	24	80	**57**	**76%**
	Female	0	0	7	66.7%	5	41.7%	6	20	**18**	**24%**
	**Total**	**12**	**100%**	**21**	**100%**	**12**	**100%**	**30**	**100%**	**75**	**100%**
**Highest level of education**	8 years of elementary school or less	5	45.5%	11	52.4%	4	36.4%	11	36.7%	**31**	**42.5%**
	Vocational school	4	36.4%	5	23.8%	4	36.4%	13	43.3%	**26**	**35.6%**
	High school	1	9.1%	4	19.0%	1	9.1%	6	20.0%	**12**	**16.4%**
	Higher education (college or university)	1	9.1%	1	4.8%	2	18.2%	0	0.0%	**4**	**5.5%**
	**Total**	**11**	**100%**	**21**	**100%**	**11**	**100%**	**30**	**100%**	**73**	**100%**
**Do you consider yourself to be a homeless person?**	No	2	18.2%	3	15.0%	4	50.0%	0	0	**9**	**13.0%**
	Yes	9	81.8%	17	85.0%	4	50.0%	30	100%	**60**	**87.0%**
	**Total**	**11**	**100%**	**20**	**100%**	**8**	**100%**	**30**	**100%**	**69**	**100%**
**How many years have you experienced homelessness?**	0–5	3	33.3%	8	47.1%	0	0,0%	8	26.7%	**19**	**31.7%**
	6–10	2	22.2%	2	11.8%	2	50.0%	14	46.7%	**20**	**33.3%**
	11–15	1	11.1%	1	5.9%	0	0,0%	5	16.7%	**7**	**11.7%**
	16 or more	3	33.3%	6	35.3%	2	50.0%	3	10.0%	**14**	**23.3%**
	**Total**	**9**	**100%**	**17**	**100%**	**4**	**100%**	**30**	**100%**	**60**	**100%**
**How often do you see a doctor/use health care services?**	Semi-annually or more frequently	10	90.9%	4	100.0%	16	55.2%	16	76.2%	**46**	**70.8%**
	Annually or less frequently	1	9.1%	0	0.0%	13	44.8%	5	23.8%	**19**	**29.2%**
	**Total**	**11**	**100%**	**4**	**100%**	**29**	**100%**	**21**	**100%**	**65**	**100%**
**Number of self-reported chronic conditions**		**mean**	**SD**	**mean**	**SD**	**mean**	**SD**	**mean**	**SD**	**mean**	**SD**
		3.58	2.15	2.67	1.20	3.50	1.45	1.90	1.16	**2.64**	**1.55**

All statistics are listed as percentage, mean or standard deviation (SD). The abbreviations for shelters are the following: *HCSOM* Hungarian Charity Service of the Order of Malta, *SHF *Shelter House Foundation, *BIC *Baptist Integration Center

### Technical feasibility and care continuity

92.2% (*n* = 415) of originally planned (*n* = 450) telecare visits took place in the community shelters as originating sites. The rate of attendance was not lower than 85% in any of the participating shelters. Overall, a total number of 55 clients (73.3%) attended all six visits, the lowest rate of full completion in shelters was 50.0%. The main reasons for missing a visit were not being in the shelter at the time of the consultation (*n* = 10 visits, 28.6%), hospitalization at the time of the consultation (*n* = 8 visits, 22.9%), and death (*n* = 5 visits – all related to the death of one client, 14.3%), while in case of 12 visits (34.3%) underlying cause was not identified. The telecare consultations lasted on an average of 12.1 min (SD = 4.07); technical problems were reported only in 7.2% (*n* = 30) of all visits. The most frequent technical problems were related to low video (*n* = 14, 48.2%) and audio (*n* = 12, 41.4%) quality. In the post-visit questionnaires, the physicians were also asked to confirm the changes in the therapeutic regime in relation to the visit and whether the parameters of chronic conditions were tracked between visits according to their expectations. They reported changes in the medication scheme in almost one-fourth of visits (*n* = 98, 23.6%) as an average for all four shelters. However, there were notable differences in the percentage of therapeutic changes between the institutions ranging from 12.4 to 60.3%. Regarding the tracking of chronic parameters, most reports stated either tracking was not necessary (*n* = 180, 43.4%) or was done according to their expectations (*n* = 189, 45.5%), and only around one-tenth of cases were where improvements would have been required. The main technical characteristics are shown in Table 2.

**Table 2:** Main technical characteristics of the pilot

Shelter		HCSOM		SHF		BIC 1		BIC 2		Total	
**Number of participants**		12		21		12		30		**75**	
		***n***	**%**	***n***	**%**	***n***	**%**	***n***	**%**	***n***	**%**
**Number of telecare consultations**	Completed	63	87.5%	112	88.9%	62	86.1%	178	98.9%	**415**	**92.2%**
	Missed	9	12.5%	14	11.1%	10	13.9%	2	1.1%	**35**	**7.8%**
	**Total planned**	**72**	**100%**	**126**	**100%**	**72**	**100%**	**180**	**100%**	**450**	**100%**
**Number of participants completed the full 6-course pilot**		8	66.7%	13	61.9%	6	50.0%	28	93.3%	**55**	**73.3%**
**Reasons of missing a consultation (number of missed visits)**	Not being in the shelter	8	88.9%	2	14.3%	0	0.0%	0	0.0%	**10**	**28.6%**
	Hospitalization	1	11.1%	7	50.0%	0	0.0%	0	0.0%	**8**	**22.9%**
	Death	0	0.0%	5	35.7%	0	0.0%	0	0.0%	**5**	**14.3%**
	Not identified	0	0.0%	0	0.0%	10	100.0%	2	100.0%	**12**	**34.3%**
	**Total**	**9**	**100%**	**14**	**100%**	**10**	**100%**	**2**	**100%**	**35**	**100%**
**Length of telecare consultations (min)**		**mean**	**SD**	**mean**	**SD**	**mean**	**SD**	**mean**	**SD**	**mean**	**SD**
		13.1	2.45	12.9	3.27	16.1	4.97	9.9	3.19	**12.1**	**4.07**
		***n***	**%**	***n***	**%**	***n***	**%**	***n***	**%**	***n***	**%**
**Did technical problems occur during the consultation?**	No	52	82.5%	108	96.4%	49	79.0%	176	98.9%	**385**	**92.8%**
	Yes	11	17.5%	4	3.6%	13	21.0%	2	1.1%	**30**	**7.2%**
	**Total**	**63**	**100%**	**112**	**100%**	**62**	**100%**	**178**	**100%**	**415**	**100%**
**Description of technical problems**	Low quality audio	0	0.0%	3	75.0%	8	66.7%	1	50.0%	**12**	**41.4%**
	Low quality video	11	100.0%	1	25.0%	1	8.3%	1	50.0%	**14**	**48.2%**
	Inaccurate document sharing	0	0.0%	0	0.0%	3	25.0%	0	0	**3**	**10.3%**
	**Total**	**11**	**100%**	**4**	**100%**	**12**	**100%**	**2**	**100%**	**29**	**100%**
**Did any change in the therapeutic regime occur during the visit?**	No	25	39.7%	92	82.1%	44	71.0%	156	87.6%	**317**	**76.4%**
	Yes	38	60.3%	20	17.9%	18	29.0%	22	12.4%	**98**	**23.6%**
	**Total**	**63**	**100%**	**112**	**100%**	**62**	**100%**	**178**	**100%**	**415**	**100%**
**Were chronic parameters tracked between the visits?**	No, but it was not necessary.	27	42.9%	20	17.9%	38	61.3%	95	53.4%	**180**	**43.4%**
	No, but it would have been useful to have the parameters tracked.	0	0.0%	1	0.9%	0	0.0%	5	2.8%	**6**	**1.4%**
	Yes, but the number of measurements has to be increased.	0	0.0%	2	1.8%	8	12.9%	30	16.9%	**40**	**9.6%**
	Yes, and the number of measurements is satisfactory.	36	57.1%	89	79.5%	16	25.8%	48	27.0%	**189**	**45.5%**
	**Total**	**63**	**100%**	**112**	**100%**	**62**	**100%**	**178**	**100%**	**415**	**100%**

All statistics are listed as percentage, mean or standard deviation (SD). During the pilot, one death occurred, and this led to 5 missed visits (client died after the first visit). The authors declare that there is no connection between the pilot program and the death. The abbreviations for shelters are the following: *HCSOM* Hungarian Charity Service of the Order of Malta, *SHF* Shelter House Foundation, *BIC* Baptist Integration Center

### Patient perspective and follow-up

The overall rating of clients regarding the telecare consultations was very high with an average of 4.52 (*n* = 415, SD = 0.68) on a 5-point Likert scale. The participants from all four shelters were consistent in this. They expressed that the on-site assistance was helpful for them (mean = 4.68, SD = 0.72), while in the applied setting, the ease of use was considered also high (mean = 4.54, SD = 0.82). According to the participants’ feedback, they could explain their complaints and medical status well (mean = 4.52, SD = 0.82), and felt that the doctor understood their problems (mean = 4.44, SD = 0.85).

After four to six months of pilot closure, a follow-up survey was also organized. 78.7% of pilot clients (*n* = 59) took part in the follow-up study. (No refusal of answering the follow-up questionnaire occurred, therefore the rate of availability was mainly determined by the change of accommodation between the pilot and the follow-up). The results showed that after four to six months, patients continued to rate past telecare consultations high (mean = 4.27, SD = 0.67) and were still willing to continue to receive this form of care (mean = 4.34, SD = 0.92). The results of the patient questionnaires and follow-up are listed in Table 3.

**Table 3:** Results of the patient questionnaires

Shelter	HCSOM		SHF		BIC 1		BIC 2		Total	
**Number of telecare visits**	63		112		62		178		**415**	
	**mean**	**SD**	**mean**	**SD**	**mean**	**SD**	**mean**	**SD**	**mean**	**SD**
**Overall, how would you rate the telemedicine visit?**	4.38	0.68	4.96	0.25	3.89	0.32	4.51	0.75	**4.52**	**0.68**
**The use of technical devices related to the telemedicine visit was easy.**	4.68	0.56	5.00	0.00	4.34	0.63	4.27	1.04	**4.54**	**0.82**
**The assistance of the on-site assistant was helpful during the telemedicine visit.**	4.79	0.45	4.96	0.30	4.97	0.18	4.35	0.94	**4.68**	**0.72**
**I was able to explain my problems well, I was able to describe my symptoms well during the telemedicine visit.**	4.49	0.86	4.99	0.09	4.36	0.68	4.29	0.98	**4.52**	**0.82**
**Based on the symptoms I described, my doctor was able to assess my condition accurately.**	4.49	0.74	4.99	0.09	3.71	0.64	4.33	0.97	**4.44**	**0.85**
**I feel that the fact that my doctor could not physically examine me, made it difficult to care.**	1.93	1.30	1.10	0.57	2.22	0.83	1.93	1.24	**1.75**	**1.12**
**I felt the telemedicine visit was comfortable.**	4.66	0.79	4.96	0.38	4.08	0.80	4.17	0.98	**4.45**	**0.88**
**The telemedicine visit took longer than an in-person visit.**	1.58	1.24	1.07	0.46	1.45	0.53	2.17	1.21	**1.68**	**1.08**
**The telemedicine visit made it difficult to communicate with my doctor.**	1.83	1.33	1.21	0.90	1.66	0.79	1.76	0.90	**1.61**	**0.99**
**I prefer an in-person visit.**	3.76	1.35	2.89	1.03	3.94	0.87	2.78	1.18	**3.13**	**1.22**
**Follow-up survey**										
**Number of pilot participants**	12		21		12		30		**75**	
	***n***	**%**	***n***	**%**	***n***	**%**	***n***	**%**	***n***	**%**
**Number of available participants during follow-up**	7	58.3%	15	71.4%	7	58.3%	30	100.0%	**59**	**78.7%**
	**mean**	**SD**	**mean**	**SD**	**mean**	**SD**	**mean**	**SD**	**mean**	**SD**
**Overall, how do you remember how you rate the telemedical consultation?**	4.29	0.76	4.60	0.51	4.00	0.58	4.17	0.70	**4.27**	**0.67**
**If telemedicine consultation became a regular service, how likely do you think you would use it?**	4.29	0.76	5.00	0.00	3.71	1.38	4.17	0.91	**4.34**	**0.92**

All statistics are listed as percentage, mean or standard deviation (SD). The abbreviations for shelters are the following: *HCSOM* Hungarian Charity Service of the Order of Malta, *SHF* Shelter House Foundation, *BIC* Baptist Integration Center

### Physician perspective and pairing of visits

The overall rating of telecare visits by the physicians was rather positive (mean = 4.79, SD = 0.65) and the average score did not fall below 4.00 for any of the shelters. The doctors felt that they were able to assess the patient’s condition properly (mean = 4.80, SD = 0.65) and make an adequate diagnosis (mean = 4.78, SD = 0.74). They felt comfortable with the telecare consultations (mean = 4.76, SD = 0.79) and did not experience major communication difficulties (mean = 1.50, SD = 0.87) (Table 4). As attitude towards in-person appointments represents an obvious comparison opportunity to traditional care pathways, data regarding this preference was further analyzed. In contrast to patients, doctors considered face-to-face visits to be significantly less important (means 1.87 vs. 3.13, *p* = 0.00).

**Table 4:** Results of the physician questionnaires

Shelter	HCSOM		SHF		BIC 1		BIC 2		Total	
**Number of telecare visits**	63		112		62		178		**415**	
	**mean**	**SD**	**mean**	**SD**	**mean**	**SD**	**mean**	**SD**	**mean**	**SD**
**Overall, how would you rate the telemedicine visit?**	4.89	0.54	4.91	0.29	4.08	1.25	4.93	0.29	**4.79**	**0.65**
**I was able to accurately assess the patient’s status based on the complaints/symptoms reported.**	4.84	0.63	4.96	0.21	4.10	1.30	4.94	0.26	**4.80**	**0.66**
**The telemedicine visit provided an adequate amount of information.**	4.79	0.68	4.92	0.27	3.87	1.41	4.90	0.35	**4.74**	**0.75**
**After the telemedicine visit, I am sure of my decision/diagnosis.**	4.75	0.69	5.00	0.00	3.90	1.41	4.96	0.33	**4.78**	**0.74**
**I felt the telemedicine visit was comfortable.**	4.75	0.82	5.00	0.00	3.84	1.45	4.92	0.39	**4.76**	**0.79**
**The telemedicine visit took longer than an in-person visit.**	1.24	0.82	1.47	0.55	1.82	1.28	1.52	1.30	**1.51**	**1.08**
**The telemedicine visit makes doctor-patient communication difficult.**	1.33	0.80	1.61	0.49	2.23	1.38	1.25	0.70	**1.50**	**0.87**
**The fact that I could not touch the patient made it difficult to make a proper diagnosis.**	1.41	0.99	1.73	0.44	2.08	1.51	1.30	0.70	**1.55**	**0.91**
**The fact that I did not see exactly the patient’s facial expression and metacommunication made it difficult to communicate.**	1.13	0.38	1.63	0.49	1.82	1.25	1.24	0.57	**1.41**	**0.71**
**I prefer an in-person visit.**	1.51	0.90	3.00	0.00	2.02	1.45	1.24	0.77	**1.87**	**1.10**

All statistics are listed as mean or standard deviation (SD). The abbreviations for shelters are the following: *HCSOM *Hungarian Charity Service of the Order of Malta, *SHF* Shelter House Foundation, *BIC* Baptist Integration Center

**Fig. 2:**
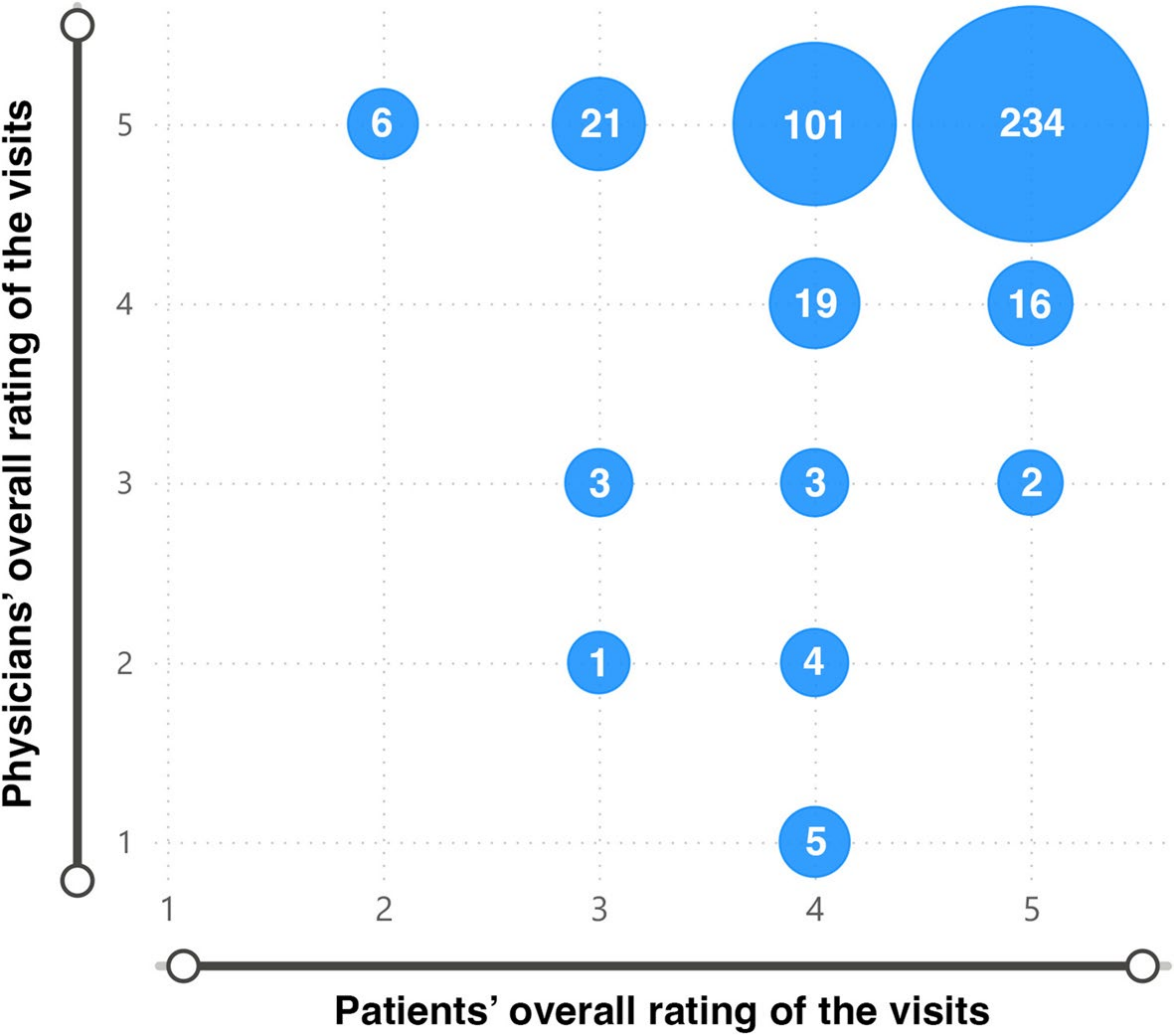
Comparison of patients’ and physicians’ overall ratings of the same visits. The ratings of patients (x-axis) and physicians (y-axis) of the same visits were paired based on a common anonymous visit ID. The figure shows the distribution of equal and alternate ratings

We were also able to pair the patient and physician reflections of the same visit through anonymous visit IDs. After pairing the patient and doctor overall ratings of each visit, an equal score from both parties was detected in 61.7% (*n* = 256) of all cases. In 30.8% (*n* = 128), the overall rating added by the physician was higher, while 7.5% (*n* = 31) of cases represented a higher score given by the patient (Fig. 2). To further analyze the occurrence of this rating discrepancy, we investigated the distribution of these alternating visits between the six appointments. In 128 visits, when a higher score was given by the doctor, a balanced distribution was seen between the rankings. 13.3% (*n* = 17) were first visits, 17.2% (*n* = 22) second, 21.1% (*n* = 27) third, 18.8% (*n* = 24) fourth, 18.0% (*n* = 23) fifth, and 11.7% (*n* = 15) sixth. However, the 31 visits, when a higher rating was given by the patient, occurred mainly during the first part of the pilot: 51.6% (*n* = 16) were first visits, 22.6% (*n* = 7) second, 19.4% (*n* = 6) third, 3.2% (*n* = 1) fourth, 3.2% (*n* = 1) fifth, and none as sixth.

### Comparison of first and sixth visits

In the next step of the analysis, the temporal score averages of the visits were compared both on the patient and physician sides. The patient sample was limited to a methodological constraint: only the responses of clients who attended all six visits were examined. This limitation was applied in order to exclude the influence of patients who rated only the initial part of the pilot but did not complete the whole course of visits. The means of patient ratings for all questions were similar or better at the sixth visit than the first. We experienced a significant difference between the first and last visits regarding the following statement *The telemedicine visit took longer than an in-person visit* (means 2.24 vs. 1.45, respectively). In the assessment of the doctors’ responses, a significant difference between their first and sixth visits was also determined for almost all statements: perceptions showed an improving trend for each item (Table 5).

**Table 5:** First and sixth visits of patients and physicians

**Patients**											
		**Overall, how would you rate the telemedicine visit?**	**The use of technical devices related to the telemedicine visit was easy.**	**The assistance of the on-site assistant was helpful during the telemedicine visit.**	**I was able to explain my problems well, I was able to describe my symptoms well during the telemedicine visit.**	**Based on the symptoms I described, my doctor was able to assess my condition accurately.**	**I feel that the fact that my doctor could not physically examine me, made it difficult to care.**	**I felt the telemedicine visit was comfortable.**	The telemedicine visit took longer than an in-person visit. *	**The telemedicine visit made it difficult to communicate with my doctor.**	**I prefer an in-person visit.**
**First visit**	*n*	55	55	55	55	55	55	55	55	55	55
	Mean	4.55	4.55	4.71	4.55	4.45	1.80	4.64	2.24	1.78	2.96
	SD	0.69	0.83	0.57	0,72	0.83	1.28	0.59	1.33	1.08	1.22
**Sixth visit**	*n*	55	55	55	55	55	55	55	55	55	55
	Mean	4.55	4.64	4.71	4.51	4.44	1.84	4.47	1.45	1.55	3.05
	SD	0.74	0.78	0.76	0.90	0.94	1.36	0.94	0.90	0.94	1.24
**Physicians**											
		Overall, how would you rate the telemedicine visit? *	I was able to accurately assess the patient’s status based on the complaints/symptoms reported. *	The telemedicine visit provided an adequate amount of information. *	After the telemedicine visit, I am sure of my decision/diagnosis. *	**I felt the telemedicine visit was comfortable.**	The telemedicine visit took longer than an in-person visit. *	The telemedicine visit makes doctor-patient communication difficult. *	The fact that I could not touch the patient made it difficult to make a proper diagnosis. *	The fact that I did not see exactly the patient's facial expression and metacommunication made it difficult to communicate. *	I prefer an in-person visit. *
**First visit**	*n*	75	75	75	75	75	75	75	75	75	75
	Mean	4.44	4.52	4.37	4.47	4.47	1.64	2.16	2.43	1.99	2.79
	SD	1.06	1.07	1.17	1.22	1.15	1.11	1.34	1.34	1.14	1.41
**Sixth visit**	*n*	55	55	55	55	55	55	55	55	55	55
	Mean	4.95	4.96	4.89	4.98	4.82	1.15	1.04	1.02	1.00	1.47
	SD	0.23	0.19	0.37	0.13	0.77	0.68	0.19	0.13	0.00	0.86

All statistics are listed as mean or standard deviation (SD). Columns marked with an asterisk (*) represent *p*<0.05

### Linear regression models

In the linear regression model of the physicians’ overall rating, the model was run in 6 steps, with the addition of a new variable in each step. The variables involved explained a significant proportion of the heterogeneity of the overall rating. Also, it reached a reasonably high explanatory rate at the 6th step (R-square = 0.632). It could be concluded from the model that there was a positive effect on the physicians’ overall rating when they were able to accurately assess the patient’s status based on the complaints/symptoms reported (Beta = 0.532). As the expansion of the model decreased the Beta value, its correlation with other variables was detected. There was also a positive effect when the doctors were sure about their decision/diagnosis after the telemedicine visit (0.231) and when there was a change in the therapeutic regime during the visit (0.134). The correlation was negative with the statement *The telemedicine visit makes doctor-patient communication difficult* (-0.148) and the length of the visit (-0.092). However, in the last step, when the variable of the agreement with the statement *The fact that I could not touch the patient made it difficult to make a proper diagnosis* entered the model, it was also positively related to satisfaction (0.102). This variable had the weakest explanatory power and the other variables also changed their Beta values. Multicollinearity could be seen here as well, and it seemed to be positively related to the doctors’ telemedicine visit rating despite the difficulty of making a diagnosis.

The linear regression model of patients’ overall rating was executed in 5 steps. It also explained significant part of the heterogeneity of the patients’ overall rating in each step, with an R-square value of 0.575 in the last step. Here, the following variables were positively related to patient satisfaction: the level of agreement with the statements *The use of technical devices related to the telemedicine visit was easy* (0.296), *Based on the symptoms reported, my doctor was able to accurately assess my condition* (0.284), and *I was able to explain my problem well* (0,227). Moreover, there was also a positive relation for the statement *The telemedicine visit took longer than an in-person visit* (0.074), and a negative correlation for *I prefer an in-personal visit* (-0.155). Both linear regression models are shown in Table 6.

**Table 6:** Linear regression models

Physicians												
**Depedent variable**: ***Overall, how would you rate the telemedicine visit?*(1–5)**												
	**Step 1**		**Step 2**		**Step 3**		**Step 4**		**Step 5**		**Step 6**	
**p**	0.000		0.000		0.000		0.000		0.000		0.000	
**Adjusted R-square**	0.588		0.604		0.615		0.625		0.628		0.632	
**Variables**	**Beta**	**p**	**Beta**	**p**	**Beta**	**p**	**Beta**	**p**	**Beta**	**p**	**Beta**	**p**
***I was able to accurately assess the patient’s status based on the complaints/symptoms reported.***	0.768	0.000	0.550	0.000	0.528	0.000	0.529	0.000	0.506	0.000	0.532	0.000
***After the telemedicine visit, I am sure of my decision/diagnosis.***			0.252	0.000	0.285	0.000	0.269	0.000	0.234	0.000	0.231	0.000
**Was there a change in the therapeutic regime the visit? (1 = no; 2 = yes)**					0.112	0.000	0.133	0.000	0.136	0.000	0.134	0.000
**Lenght of consultation (min)**							-0.106	0.001	-0.088	0.007	-0.092	0.004
***The telemedicine visit makes doctorpatient communication difficult.***									-0.092	0.024	-0.148	0.002
***The fact that I could not touch the patient made it difficult to make a proper diagnosis.***											0.102	0,019
**Patients**												
**Depedent variable**: ***Overall, how would you rate the telemedicine visit?*(1–5)**												
			**Step 1**		**Step 2**		**Step 3**		**Step 4**		**Step 5**	
**p**			0.000		0.000		0.000		0.000		0.000	
**Adjusted R-square**			0.445		0.521		0.549		0.566		0.570	
**Variables**			**Beta**	**p**	**Beta**	**p**	**Beta**	**p**	**Beta**	**p**	**Beta**	**p**
**Based on the symptoms I described, my doctor was able to assess my condition accurately.**			0.668	0.000	0.398	0.000	0.348	0.000	0.278	0.000	0.284	0.000
**The use of technical devices related to the telemedicine visit was easy.**					0.387	0.000	0.389	0.000	0.293	0.000	0.296	0.000
**I prefer an in-person visit.**							-0.175	0.000	-0.158	0.000	-0.155	0.000
**I was able to explain my problems well, I was able to describe my symptoms well during the telemedicine visit.**									0.208	0.000	0.227	0.000
**The telemedicine visit took longer than an in-person visit.**											0.074	0.035

## Discussion

People experiencing homelessness seek acute hospital-based and emergency care at a higher rate compared to the general population [31–33], while they report several barriers to regular chronic care [34–37]. As digital health technologies tend to form an increasing part of primary care, online platforms might represent both additional access and barrier for vulnerable populations [12, 38, 39]. Despite this notable discrepancy, telehealth tools and interventions are much less studied in these populations [40, 41]. In order to investigate the feasibility and effectiveness of telehealth among people experiencing homelessness in Hungary, our research team completed a pilot study focusing on the management of chronic conditions. This provides unique insight regarding the implementation of telecare in a social care institutional environment.

Telehealth services were based on synchronous online video consultations with a physician. Study participants were present on more than 90% of initially planned visits and almost three-quarters of recruited clients completed the whole course of six online visits. This supports the significant potential of telecare services in catalyzing care continuity among people experiencing homelessness. Results from both the patient and physician questionnaires further emphasize the viability of such services. The overall client satisfaction was high, and participants reported similarly high ratings for ease of use and comfort. This positive attitude remained consistent in the follow-up questionnaires with a notable openness towards a regular telecare service. From the physicians’ perspective, the overall rating was also prominent with high scores regarding the ability to assess the patients’ health status, the amount of available information, and comfort of service. These results are similar to the ones reported by a study from the USA investigating the user and provider experience of telemedicine among people experiencing homelessness [28].

Furthermore, a significant improvement was also detected in the physicians’ questionnaires regarding almost all aspects between their first and sixth visits. These results possibly support the phenomenon of acquiring a telecare routine in such a short term and/or refinement of their aims and expectations with time. After pairing the overall ratings of clients and doctors for each visit, these possibilities were also supported by the result that visits, where the patients’ overall score was higher than the physicians’, occurred mostly during the first part of the pilot. In addition, the overall rating of patients and physicians was equal on a 5-point scale in more than 60% of all cases, indicating that there was no notable perception bias on the originating and receiver sites during the majority of visits. However, there was a significant difference in the reactions of clients and doctors to the *I prefer an in-person visit* statement. A possible explanation might be that physicians were satisfied with the level of effectiveness of telecare from a professional point of view and targeted use of this service provided similar results as in-person appointments would have. Clients, who encountered telemedicine first in this pilot, faced a new kind of care that lacked physical contact and this pilot was not able to change their basic concept of medical service.

According to the post-visit questionnaires of the physicians, almost one-fourth of visits led to a therapeutic modification in such a short time frame. This underpins that new care pathways might reach patients who were previously excluded from continuous medical supervision. These reports also strengthened the feasibility of our telecare setup by showing a high rate of compliance in providing appropriate chronic parameter measurements between visits to support the medical decision-making process. We believe this was also catalyzed by the on-site assistants serving as a valuable aid not only during the visits but between appointments as well.

Our linear regression models proved that the accurate assessment of the patient’s health status and confidence in the diagnosis had the most important positive effects on the overall satisfaction of physicians, while other variables (therapeutic change, communication difficulties, length of the visit) also affected that. In the patient linear regression model, the influence of a couple of variables (ease of use, confidence in the doctor’s assessment, lack of communication gaps, and preference for an in-person visit) was also detected.

This pilot represented the first multicentric telehealth activity of the Health Center at the Hungarian Charity Service of the Order of Malta. Due to inexperience in the field, a number of study design decisions had to be made. The pilot involved assistants on the originating site who were present during the online visits together with the patients. Although this could raise privacy issues, the research team applied this decision based on two major reasons. First, this form of a care team is the same setup as usually seen during a normal in-person primary care appointment in Hungary when both the primary care physician and the practice nurse are present during every visit. Second, according to our past experiences in the field of medical care of people experiencing homelessness, most of our patients are struggling with digital and health literacy deficits. The presence of an on-site assistant both served technical support and prevented any misunderstandings regarding medication or referral issues. The patients also reported in the questionnaires that this kind of assistance was very helpful during the telecare visits. A review published after this decision was made also found that trusted intermediaries play an important role in telehealth delivery [42].

Another potential privacy issue related to telemedical care is the choice of communication channels. Although study participants used anonymized accounts, utilizing widely available, popular video call services, this was considered a security risk. However, at the outbreak of the COVID-19 pandemic, applying these services for telemedicine was supported by the American Medical Association and used in other studies as well [43, 44]. As a further benefit, these platforms might have been familiar for the digitally engaged clients providing more confidence to them during the telecare visits. But on a longer term, specific, user-friendly, and secure online telemedicine software solutions have to be implemented into the telecare workflow.

In our follow-up study, the fluctuation among residents reached more than 40% in two of the participating communal shelters in a four to six months’ timeframe even though these institutions offered mid- and long-term accommodation to people experiencing homelessness. In case of a future service extension towards night shelters and other types of social services (e.g., soup kitchens, day centers, emergency shelters), where fluctuation is expected to be much higher, telemedicine should be organized through a coordinated telecare network of local social and healthcare providers. Only this could guarantee continuous availability for clients irrespectively to their actual accommodation or shelter organization and prevent the drop-out of a patient after moving into a different shelter or housing solution.

Although, during the preparation phase, focus group discussions included both physicians and on-site assistants, lacked the client perspective. When telehealth offerings become a regular service, due to the Collaborative and Community-Driven Research principles, homeless clients should be also involved in the fine-tuning process of the model.

Our study had certain limitations. Research focusing on people experiencing homelessness is usually limited in the number of participants and this limitation is represented in our pilot program as well. A further limitation is that our participants were recruited exclusively from communal shelters providing mid- and long-term accommodation therefore we have excluded well-characterized subpopulations such as rough sleepers or clients frequently changing their accommodation. As participation in the pilot was voluntary, homeless persons with a more positive attitude and openness towards telecare might have been overrepresented in our sample and the results of the study might overestimate the general telecare compliance of people experiencing homelessness. Also, we have limited information on the reasons for missing a visit. The extension of the telecare service would clearly benefit from the feedback of clients who have stopped attending the appointments at some time. Finally, we were also focusing only on the telemedical management of chronic conditions of our participants. Acute illnesses and response to emergency situations through telehealth services were not included in the pilot.

## Conclusion

In conclusion, our study provided evidence of a feasible telecare setup in shelters offering accommodation to people experiencing homelessness. During the pilot, we measured numerous patient and provider aspects after every online session throughout the whole length of the study and reached an extremely high level of data integrity. Our results emphasized that a group of previously digitally excluded homeless persons found the telecare visits useful and valuable, while the physicians reported high medical relevance in chronic care. These experiences might support the planning of future telehealth services for vulnerable populations.

## Supplementary Information

**Additional file 1.** Questionnaires. All questionnaires used in the study are available both in English and in Hungarian.

## Data Availability

The datasets generated during the current study are available from the corresponding author on reasonable request.
