# Supplementary material for: Telemedicine in community shelters: possibilities to improve chronic care among people experiencing homelessness in Hungary
Source: Int J Equity Health. 2022 Dec 17;21:181. doi: 10.1186/s12939-022-01803-4 (PMC9758475; doi:10.1186/s12939-022-01803-4)
Supplement: Supplementary file 1 — Additional file 1. Questionnaires. All questionnaires used in the study are available both in English and in Hungarian. [file 12939_2022_1803_MOESM1_ESM.pdf]

Supplementary Materials

Békási S, Girasek E, Győrffy Z. Telemedicine in community shelters: possibilities to improve chronic care among people experiencing homelessness in Hungary.

This file contains the following materials:

Patient questionnaire in English
Patient follow-up questionnaire in English
Physician questionnaire in English
Patient questionnaire in Hungarian (original)
Patient follow-up questionnaire in Hungarian (original)
Physician questionnaire in Hungarian (original)

By sharing these supplementary materials, the authors would like to support the reproducibility of the study and provide an additional context for their research.

1. Patient questionnaire in English

Overall, how would you rate the telemedicine visit?

	1	2	3	4	5	
Very bad	☐	☐	☐	☐	☐	Very good

Description of technical problem (if any):

....

STATEMENT	ANSWER (Check the most appropriate box)				
	Not true	Slightly true	Somewha t true	Mostly true	Absolutely true
The use of technical devices related to the telemedicine visit was easy.	☐	☐	☐	☐	☐
The assistance of the on-site assistant was helpful during the telemedicine visit.	☐	☐	☐	☐	☐
I was able to explain my problems well, I was able to describe my symptoms well during the telemedicine visit.	☐	☐	☐	☐	☐
Based on the symptoms I described, my doctor was able to assess my condition accurately.	☐	☐	☐	☐	☐
I feel that the fact that my doctor could not physically examine me, made it difficult to care.	☐	☐	☐	☐	☐
I felt the telemedicine visit was comfortable.	☐	☐	☐	☐	☐
The telemedicine visit took longer than an in-person visit.	☐	☐	☐	☐	☐
The telemedicine visit made it difficult to communicate with my doctor.	☐	☐	☐	☐	☐
I prefer an in-person visit.	☐	☐	☐	☐	☐

2. Patient follow-up questionnaire in English

Overall, how do you remember how you rate the telemedical consultation?

☐ Very bad

☐ Bad

☐ Medium

☐ Good

☐ Very good

If telemedicine consultation became a regular service, how likely do you think you would use it?

☐ Not at all

☐ Maybe

☐ Moderately

☐ Probably

☐ Definitely

3. Physician questionnaire in English

Length of consultation: ……………………… min

Has the patient had regular measurements of any medical parameters since the last consultation?

□ No, and it wasn't necessary.
□ No, but it would have been necessary.
□ Yes, but the number of measurements is less than required.
□ Yes, and a sufficient number of measurements is available.

Was there a change in therapy recommendations during the consultation?

□ Yes. □ No.

Overall, how would you rate the telemedicine visit?

	1	2	3	4	5	
Very bad	□	□	□	□	□	Very good

Did you experience any technical problems during the consultation?

□ No.
□ Yes, in the quality of the audio.
□ Yes, in the quality of the video.
□ Yes, regarding the document sharing.
□ Yes, the area / body part to be examined was not clearly visible.
□ Yes, other: ……………………………………………………………………………………………

**STATEMENT**	**ANSWER (Check the most appropriate box)**				
	**Not true**	**Slightly true**	**Somewhat true**	**Mostly true**	**Absolutely true**
I was able to accurately assess the patient's status based on the complaints/symptoms reported.	□	□	□	□	□
The telemedicine visit provided an adequate amount of information.	□	□	□	□	□
After the telemedicine visit, I am sure of my decision/diagnosis.	□	□	□	□	□
I felt the telemedicine visit was comfortable.	□	□	□	□	□
The telemedicine visit took longer than an in-person visit.	□	□	□	□	□
The telemedicine visit makes doctor-patient communication difficult.	□	□	□	□	□
The fact that I could not touch the patient made it difficult to make a proper diagnosis.	□	□	□	□	□
The fact that I did not see exactly the patient's facial expression and metacommunication made it difficult to communicate.	□	□	□	□	□
I prefer an in-person visit.	□	□	□	□	□

4. Patient questionnaire in Hungarian (original)

Összességében hogyan értékelné a telemedicinális vizitet?

	1	2	3	4	5	
Nagyon rossz	☐	☐	☐	☐	☐	Nagyon jó

Technikai probléma leírása (ha volt):

....

**ÁLLÍTÁS**	**VÁLASZ (Jelölje meg a megfelelő mezőt)**				
	**Nem igaz**	**Kevéssé igaz**	**Valamennyire igaz**	**Többnyire igaz**	**Teljesen igaz**
A távvizittel kapcsolatos technikai eszközök használata egyszerű volt.	☐	☐	☐	☐	☐
Az intézményi segítő szakember (asszisztens) segítsége hasznos volt a távvizit során.	☐	☐	☐	☐	☐
Jól el tudtam magyarázni a problémámat, jól le tudtam írni a tüneteimet a távvizit során.	☐	☐	☐	☐	☐
Az elmondott tünetek alapján az orvosom pontosan fel tudta mérni az állapotomat.	☐	☐	☐	☐	☐
Úgy érzem, hogy az, hogy fizikailag nem tudott megvizsgálni az orvosom, megnehezítette az ellátást.	☐	☐	☐	☐	☐
A távvizitet kényelmesnek éreztem.	☐	☐	☐	☐	☐
A távvizit hosszabb időt vett igénybe, mint a személyes találkozó.	☐	☐	☐	☐	☐
A távvizit megnehezítette az orvosommal való kommunikációt.	☐	☐	☐	☐	☐
A személyes vizitet jobban kedvelem.	☐	☐	☐	☐	☐

5. Patient follow-up questionnaire in Hungarian (original)

Összességében hogyan emlékszik vissza, hogyan értékeli az online orvosi konzultációt?

☐ Nagyon rossz

☐ Rossz

☐ Közepes

☐ Jó

☐ Nagyon jó

Ha rendszeres szolgáltatássá válna az online orvosi konzultáció, mennyire tartja valószínűnek, hogy igénybe venné?

☐ Egyáltalán nem

☐ Talán

☐ Közepesen

☐ Valószínűleg igen

☐ Egészen biztosan

6. Physician questionnaire in Hungarian (original)

A vizit időtartama: ……………………… perc

Történt rendszeres orvosi eszközös mérés az előző vizit óta?

□ Nem, de nem is volt rá szükség.
□ Nem, pedig szükség lett volna rá.
□ Igen, de a mérési adatok száma kevesebb, mint ami szükséges.
□ Igen, és elegendő mérési adat áll rendelkezésre.

Történt-e terápiamódosítás a vizit alatt?

□ Igen. □ Nem.

Összességében hogyan értékelné a telemedicinális vizitet?

	1	2	3	4	5	
Nagyon rossz	□	□	□	□	□	Nagyon jó

Tapasztalt-e bármilyen technikai jellegű problémát a távvizit alatt?

□ Nem.
□ Igen, a hangminőségben.
□ Igen, a képminőségben.
□ Igen, a dokumentumok megosztásával kapcsolatban.
□ Igen, nem volt jól látható a megvizsgálandó terület/testrész.
□ Igen, másban: ……………………………………………………………………………………………

ÁLLÍTÁS	VÁLASZ (Válassza a legmegfelelőbb mezőt)				
	Nem igaz	Kevéssé igaz	Valamennyire igaz	Többnyire igaz	Teljesen igaz
Az elmondott panaszok/tünetek vagy a beteg beszámolója alapján pontosan fel tudtam mérni a helyzetet.	□	□	□	□	□
A telemedicinális vizit megfelelő információmennyiséget nyújtott.	□	□	□	□	□
A távvizit után biztos vagyok a döntésemben/diagnózisomban.	□	□	□	□	□
A távvizitet kényelmesnek éreztem.	□	□	□	□	□
A távvizit hosszabb időt vett igénybe, mint a személyes találkozó.	□	□	□	□	□
A távvizit megnehezíti az orvos-beteg kommunikációt.	□	□	□	□	□
Az, hogy nem tudtam megérinteni a pácienst, megnehezítette a diagnózis kialakítását.	□	□	□	□	□
Az, hogy nem láttam pontosan a páciens arckifejezését, metakommunikációját, megnehezítette a kapcsolattartást.	□	□	□	□	□
A személyes vizitet jobban kedvelem.	□	□	□	□	□
